# A two year randomized clinical trial comparing opposing enamel wear from milled resin-matrix ceramic and direct bulk-fill composite overlays

**DOI:** 10.1038/s41405-026-00400-9

**Published:** 2026-02-21

**Authors:** Esraa Esmeail H. Elhaddad, Aly Ayman M. Elkady, Dina Fayez S. Diab

**Affiliations:** 1 Conservative Dentistry Department, Faculty of Dentistry, Misr University for Science and Technology, Cairo, Egypt; 2 Fixed Prosthodontics Department, Faculty of Dentistry, Misr International University, Cairo, Egypt; 3 Conservative Dentistry Department, Faculty of Dentistry, Egyptian Russian University, Cairo, Egypt

**Keywords:** Bonded restorations, Minimal intervention dentistry, Fixed prosthodontics

## Abstract

**Background:**

Tooth wear has become an increasingly prevalent clinical issue. It can lead to a reduction in the vertical dimension of occlusion (VDO), heightened tooth sensitivity, and alterations in both function and esthetics due to the progressive loss of dental hard tissues. As the incidence and severity of this multifactorial condition continue to rise, there is a growing need for dentists to adopt minimally invasive approaches for diagnosis, monitoring, and management. This clinical trial was designed to evaluate the amount of enamel wear on opposing enamel in response to either indirect milled resin matrix ceramic or direct bulk-fill resin composite overlays, using intraoral digital scanning technology.

**Methods:**

Eleven patients in each group received treatment for a total of 22 restorations. Participants were split into two groups, with R1 representing endodontically treated teeth restored with milled resin matrix ceramic overlays and R2 representing endodontically treated teeth restored with direct bulk-fill composite overlays. Using intraoral scanners and three-dimensional surface-based superimposition software, tooth enamel wear was assessed intraorally at baseline (T0), 12 months (T12), and 24 months (T24). The statistical analyses conducted included the Shapiro–Wilk test and the Mann–Whitney *U* test. The significance level was set at *p* < 0.05 across all tests.

**Results:**

The mean linear wear ( ± SD) for the indirect restorations was 0.41 ± 0.27 mm, compared to 0.20 ± 0.05 mm for the direct restorations; this difference was not statistically significant (*p* = 0.066). Similarly, the mean volumetric wear was 0.13 ± 0.06 mm³ for the indirect group and 0.12 ± 0.08 mm³ for the direct group, with no significant difference between them (*p* = 1).

**Conclusions:**

Within the limitations of this 2-year clinical evaluation, both direct bulk-fill resin composites and milled resin matrix ceramic overlays demonstrated comparable behavior with opposing enamel in posterior teeth, with no statistically significant differences between the tested materials.

**Trial registration:**

The study was registered at ClinicalTrials.gov on 29 Jan 2025 (#NCT06807125).

Tooth wear is a growing concern these days. The loss of tooth hard tissue is caused by a variety of reasons. Prior studies [1, 2] have demonstrated that the three main causes of worn dentition abrasion, attrition, and erosion/biocorrosion display reciprocal interactions. Reduced vertical dimension of occlusion (VDO), increased tooth sensitivity, and changed function and appearance can all be consequences of an accelerated loss of hard tissue. In order to respond as fast and effectively as possible while utilizing the least invasive techniques, dentists must develop new methods for diagnosis, patient monitoring, and treatment due to the growing incidence and severity of this complex issue [3].

Traditional treatment methods that employ metal-based crowns and fixed dental prostheses (FDPs) are currently considered the gold standard for clinical survival and success. The substantial amount of tooth structure that needs to be removed in order to prepare the crown and abutment is one disadvantage of these restorations [4].

Milled resin matrix ceramics also have certain advantages. These include more material flexibility in comparison to ceramics and less invasiveness due to the superior properties of milled resin matrix ceramics. The characteristics of the polymeric material and its exceptional edge stability enable treatments with very thin layers of the material and minimal depth of preparation margins, or maybe no preparation at all, to optimize tooth conservation. Additionally, it has been seen that milled resin matrix ceramic restorations exhibit better wear behavior on the opposing enamel, which is advantageous from a biomimetic standpoint because the tooth’s characteristics would be preserved [5].

The clinical long-term of direct and indirect resin composite restorations is comparable for short-, medium, and long-term observation periods, according to Josic et al. 2023 systematic review and meta-analyses, which have a very low certainty of evidence. Furthermore, it appears that the short-term survival of resin composite materials applied to posteriorly worn teeth is unaffected by the restorative technique selection. More long-term RCTs are required to validate the results of the current systematic review, according to the observed quality of the evidence [6].

A lack of a gold standard reference frequently prevented the validity of other highly repeatable quantitative methods, including digital profilometry and 3D surface matching techniques, from being assessed. Additionally, some methods are too complicated to be applied in clinical practice since they require specific tools, imprints, and the construction of physical models. As imaging techniques become more accurate and cost-effective over time, any quantitative methodology that uses digital models at any stage will be more applicable. In previous studies, we suggested accurate 3D superimposition methods to quantify tooth wear on serial digital dental models created intraorally, which might be applied in many clinical contexts. Since intraoral scanners have become widely employed in modern dentistry, these techniques can be easily applied in clinical settings with the right software [7].

This clinical trial was conducted to measure the intraoral wear of opposing enamel against two types of posterior overlays indirect milled resin-matrix ceramic and direct bulk-fill resin composite using a digital scanner. Despite their common clinical application, comparative data on their long-term effects on opposing enamel wear are limited, justifying this investigation. The null hypothesis was that no statistically significant difference in enamel wear would be found between the two types of overlays in the posterior region.

## Materials and methods

### Study design

This study was a randomized clinical trial (RCT) with a parallel-arm design and an equivalence framework, conducted to measure the amount of wear of the opposing enamel to milled resin matrix ceramic versus direct bulk fill resin composite overlays in posterior teeth after 2 years of performance. The allocation ratio was 1:1. The study was double-blinded, involving blinding of both the patient and the outcome assessor, while blinding the operator was not feasible due to the differing application techniques of the restorative materials. This clinical trial was held in Soad Kafafy University Hospital, Clinics of the Faculty of Dentistry, and other core labs in MUST University. The protocol was approved by the ethical committee of MUST University (2024/0063) and registered at ClinicalTrials.gov on 29 Jan 2025 (#NCT06807125). Patients signed the informed consent after receiving an oral explanation.

### Sample size calculation

A power analysis was designed to have adequate power to apply a two-sided statistical test of the null hypothesis that no difference would be found between different tested groups regarding opposing enamel wear. By adopting alpha (α) and beta (β) levels of (0.05) (i.e., power = 95%), and an effect size (d) of) 1.84) calculated based on the results of a previous study [8], the total required sample size (n) was found to be (18) cases (9 cases per group). The sample size was increased to account for possible dropouts during different follow up intervals to be (22) cases (i.e., 11 cases per group). Sample size calculation was performed using R statistical analysis software version 4.4.1 for Windows [9].

### Randomization and blinding

**Fig. 1:**
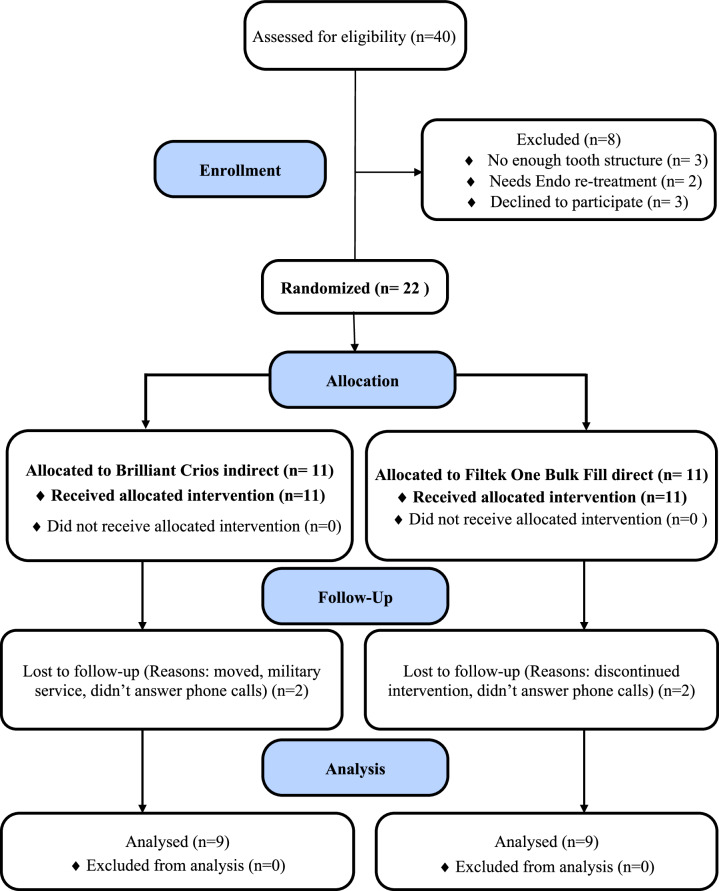
Flowchart illustrating patient enrolment, allocation, follow-up and analysis (*n* = number).

Patients were recruited until reaching the target sample size from the outpatient clinic of Soad Kafafy University Hospital, Clinics of Faculty of Dentistry in MUST University. The medical and dental history of each patient were acquired. Diagnostic and examination charts were completed. To verify the periapical condition of the involved teeth, a radiographic examination was also conducted. All participants received oral hygiene education, and any additional dental concerns were addressed. Following the intervention, patients were told not to use abrasive toothpaste or hard toothbrushes. Using Random Sequence Generator, Randomness and Integrity Services Ltd., a web-based application (www.randomization.com), contributors carried out simple randomization by creating random numbers. Every randomly generated number between 1 and 11 denotes the intervention, while 12–22 denotes the comparator. An opaque sealed envelope containing random numbers was assigned by the prior contributor, who was no longer involved in the clinical trial’s future phases. The operator recorded the outcomes of the randomization process on a computer and retained all of the records. The flow of participants through each stage of the trial was summarized in the CONSORT diagram (Fig. 1).

### Patient selection and eligibility criteria

a-Eligibility Criteria of participants:

#### Inclusion Criteria of participants

Male or female patients aged 18 to 55 with severely decayed first or second molar who also had adequate dental hygiene and consented to participate in the trial.

#### Exclusion criteria of participants

The patients had a history of severe medical difficulties, including pregnancy, xerostomia, temporomandibular joint abnormalities, a history of methacrylate allergies, parafunctional behaviors, lack of cooperation, and excessive smoking.

b-Eligibility Criteria of teeth:

#### Inclusion criteria of teeth

Endodontically treated upper or lower molar teeth with a consistent root canal filling that terminates 1–2 mm from the radiographic apex and is asymptomatic.

#### Exclusion criteria of the teeth

Teeth that may eventually require prosthodontic therapy, have deep subgingival cavity borders, and have serious periodontal problems.

### Preoperative assessment

Prior to enrollment and randomization, all potential participants underwent a comprehensive preoperative assessment. Occlusal relationships were evaluated in both centric relation and maximum intercuspation to identify any pre-existing deflective contacts, interferences, or occlusal discrepancies. This assessment was performed clinically using Shimstock foil and articulating paper.

A thorough screening for parafunctional habits (e.g., bruxism, clenching) was conducted through a structured patient interview. This interview specifically inquired about awareness of grinding or clenching teeth during the day or night, as reported by a sleep partner, and the presence of common clinical signs such as morning masticatory muscle fatigue or tenderness. As per the exclusion criteria, patients with a positive history of significant parafunctional habits were not included in the study to minimize confounding factors on wear measurements.

### Clinical procedure

Participants were randomly allocated to one of two test groups: the Intervention Group (G1), which received indirect milled resin-matrix ceramic overlays (BRILLIANT Crios), and the Comparator Group (G2), which received direct bulk-fill resin composite overlays (Filtek™ One Bulk Fill). The detailed composition and specifications of these restorative materials are provided in Table 1. A standardized overlay preparation protocol was followed for all teeth in both groups. Standardized cavity preparation was performed on both groups. After local anesthesia was administered, the tooth was isolated using a rubber dam. An occlusal reduction of 1.5 mm on functional cusps and 1.0 mm on non-functional cusps was performed using a wheel stone positioned parallel to the occlusal plane and aligned with the long axis of the tooth. This approach facilitated the creation of a supragingival butt-joint margin while maintaining adequate enamel thickness to optimize adhesive effectiveness. A composite build-up was subsequently carried out employing Immediate Dentin Sealing (IDS) and, when indicated, Cervical Margin Relocation (CMR). Thereafter, the cavity was prepared and refined according to the principles of the modified morphology-driven preparation technique (MDPT) [10].

**Table 1:** Composition and specifications of the restorative materials used in the study.

Material Type	Manufacturer	Material Classification/Composition	LOT Number
Indirect Milled Resin-Matrix Ceramic	BRILLIANT Crios (Coltène, Altstätten, Switzerland)	Organic Matrix: Dimethacrylates (20–25 wt%). Inorganic Fillers: Silanized ceramic (Barium glass, SiO₂) and ytterbium trifluoride (75–80 wt%). Filler Size: 0.1–3.0 µm. Filler Loading: ~77 wt%, ~58 vol%.	K35262
Direct Bulk-Fill Resin Composite	Filtek™ One Bulk Fill (3 M ESPE, St. Paul, MN, USA)	Organic Matrix: AUDMA, UDMA, 1,12-dodecane-DMA. Inorganic Fillers: Silanized fillers (Zirconia/Silica clusters), non-agglomerated silica nanoparticles (55–60 wt%). Filler Size: 20 nm to 3.0 µm (cluster size). Filler Loading: ~76.5 wt%, ~58.4 vol%.	NE09753

*wt%* weight percentage, *vol%* volume percentage, *AUDMA* Aromatic Urethane Dimethacrylate, *UDMA* Urethane Dimethacrylate.

### For the intervention group (G1)

After intraoral scan taking, a light-cured provisional restoration was fabricated. The definitive overlays were milled from BRILLIANT Crios (Coltène, Altstätten, Switzerland). The intraoral scan data were used to digitally design the overlay using the CAD software of the Sirona MC X5 system (Dentsply Sirona). The design file was then transferred to the integrated Sirona MC X5 milling unit for fabrication. Before proceeding with the cementation, the fit of the restoration, including marginal adaptation and proximal contacts, was thoroughly assessed. The internal surface of the overlays underwent air abrasion using 50 µm aluminum oxide particles, followed by rinsing, drying, application of an adhesive agent, and light curing for 20 s. The tooth surface was air-abraded using 50 µm particles, etched for 15 s, rinsed for an additional 15 s, and gently air-dried. An adhesive system was then applied and light-cured for 20 s. Finally, the overlay was cemented using a preheated composite resin.

### For the comparator group (G2)

Following the standardized tooth preparation, a putty index was fabricated from a diagnostic wax-up. This wax-up was created on a study model to anatomically contour the missing buccal and lingual walls, re-establishing the core tooth structure up to the midpoint of the cusp tips. The tooth was then isolated with a rubber dam. The tooth surface was etched for 15 s, rinsed for another 15 s, and gently air-dried. An adhesive was applied and light-cured for 20 seconds.

The restorative build-up commenced with the reconstruction of the buccal and lingual walls. Using the sectioned putty index as a guide, the walls were built up with Filtek™ One Bulk Fill (3 M, USA) to a height corresponding to the midpoint of the cusp tips, re-establishing the core tooth structure. Proximal walls were restored using sectional matrices, diamond-shaped wedges, and Garrison rings. Finally, the occlusal portion was built up to reestablish the full anatomical contours and functional integrity. Each increment was light-cured for 20 s. Occlusal contacts were adjusted using articulating paper, and the procedure concluded with polishing and finishing [11].

### Measurement of opposing enamel wear

Patients were scanned at three distinct intervals: immediately following restoration placement (T0), and subsequently at 12 months (T1), and 24 months (T2).

### Wear quantification process

#### Data collection

After completing adhesive bonding and occlusal adjustments, the dentition was isolated using lip retractors and cotton rolls. The occlusal surfaces were thoroughly air-dried to eliminate any residual moisture. The opposing tooth to the restoration, along with its adjacent mesial and distal teeth, was scanned at baseline and during follow-up appointments using an intraoral scanner (Panda 2; SHINING 3D) adhering strictly to the manufacturer’s recommended scanning protocol.

#### Dataset processing

STL files obtained from baseline and follow-up scans were imported into Geomagic Control X 2022, a 3D metrology software. The digital models were segmented to isolate the opposed molars to restorations, which were then saved independently to facilitate targeted analysis of wear associated with the opposed enamel.

#### Superimposition of data

**Fig. 2:**
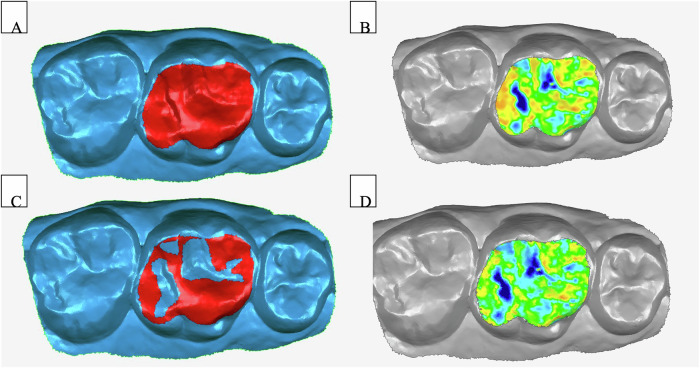
Iterative alignment and linear deviation measurement. **A** Initial region for alignment and comparison. **B** Heatmap result of initial alignment. **C** Refined selection for iterative alignment. **D** Heatmap result of deviation after refined alignment.

Recall scan data were superimposed onto baseline datasets using an iterative best-fit alignment technique. This method employed algorithms to progressively align the pre- and post-wear models, utilizing stable, unaltered tooth surfaces as reference areas to minimize alignment discrepancies. Visual inspection was conducted to evaluate the accuracy of the initial superimposition, followed by refined alignment steps that excluded regions exhibiting significant deviations. This process was repeated until no further improvement in alignment accuracy was achieved. Only datasets demonstrating an overlay error of less than 0.03 mm were included in the final analysis. The iterative alignment facilitated precise localization of wear zones on the opposed enamel. Superimposed models were visualized as color-coded heatmaps, with worn regions appearing in blue to denote areas of opposing-induced wear. A representative example of this iterative alignment process and the resulting heatmap is provided in Fig. 2.

##### Linear deviation measurement

Linear deviation was assessed to quantify material loss or depth of wear between two 3D datasets. The 3D comparison tool was used to highlight the occlusal surface for evaluation, and the maximum negative deviation value was recorded.

##### Volumetric deviation measurement

**Fig. 3:**
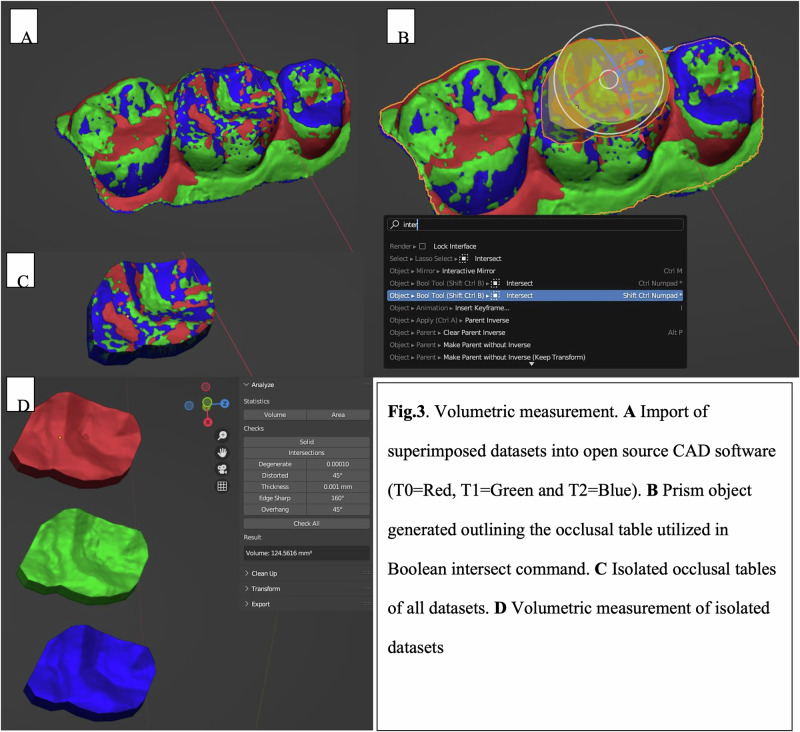
Volumetric measurement. **A** Import of superimposed datasets into open source CAD software, T0 = Red, T1 = Green, T2 = Blue. **B** Prism object generated outlining the occlusal table utilized in Boolean intersect command. **C** Isolated occlusal tables of all datasets. **D** Volumetric measurement of isolated datasets.

Volumetric deviation analysis was used to determine the total material loss between two 3D datasets. The aligned datasets were exported from the metrology software, retaining their adjusted coordinates, and imported into an open-source 3D modeling software (Blender v3.9 LTS). The sequential steps of this volumetric measurement process, from dataset import to final volume calculation, are detailed in Fig. 3. A prism object intersecting the occlusal table was created based on the occlusal surface morphology. The Boolean intersect command was then used to isolate the occlusal table. The “3D Print Toolbox” add-on was used to measure the volume of the isolated occlusal table at baseline and at the T2 follow-up. The volume difference, calculated in cubic millimeters (mm³), was recorded in a results spreadsheet.

##### Outcome measures and data compilation

The recorded linear and volumetric measurements for all 18 patients were compiled for statistical analysis. Descriptive statistics were calculated, and the changes in wear depth and volume loss between the baseline (T0) and the 24-month (T2) follow-up were analyzed to compare the two restorative groups.

#### Statistical analysis

Numerical data (deviation data) were presented as mean, standard deviation (SD), median and interquartile range (IQR) values. They were examined for normality by assessing the distribution as well as using the Shapiro–Wilk’s test and were found to be non-parametric. They were analyzed using the Mann–Whitney *U* test. The significance level was set at *p* < 0.05 within all tests. Statistical analysis was performed with R statistical analysis software version 4.4.2 for Windows (R Core Team (2024). R: A language and environment for statistical computing. R Foundation for Statistical Computing, Vienna, Austria. URL https://www.R-project.org/).

### Ethics approval and consent to participate

This study was conducted in full accordance with the ethical principles, including the World Medical Association Declaration of Helsinki, and authorized by Institutional Review Board at Faculty of Density, Misr University for Science and Technology University, Egypt on December 4 2024 (2024/0063).

## Results

**Fig. 4:**
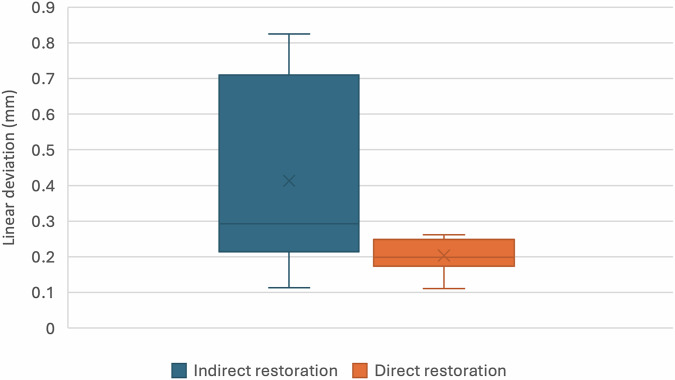
Box plot for linear deviation values.

**Fig. 5:**
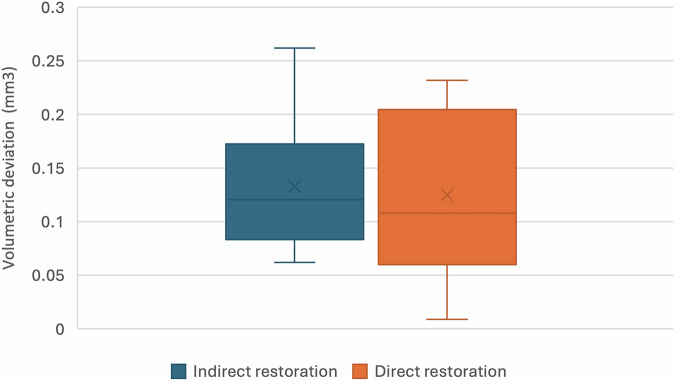
Box plot for volumetric deviation values.

The results of deviation measurements for opposing enamel wear over the two-year study period are presented in Table 2 and illustrated in Figs. 4 and 5. For linear wear depth, the mean ( ± SD) values were 0.41 ± 0.27 mm for the indirect restorations (BRILLIANT Crios) and 0.20 ± 0.05 mm for the direct restorations (Filtek™ One Bulk Fill). The difference between the groups was not statistically significant according to the Mann–Whitney *U* test (*U* = 68.00, *p* = 0.066).

**Table 2:** Deviation measurements.

Parameter		Indirect restoration	Direct restoration	Tests statistics	*p*-value
Linear deviation (mm)	Mean ± SD	0.41 ± 0.27	0.20 ± 0.05	68.00	0.066
	Median (IQR)	0.29 (0.40)	0.20 (0.05)		
Volumetric deviation (mm^3^)	Mean ± SD	0.13 ± 0.06	0.12 ± 0.08	45.00	1
	Median (IQR)	0.12 (0.07)	0.11 (0.11)		

The volumetric wear analysis is presented in Table 2 and illustrated in Fig. 5. The mean ( ± SD) volumetric deviation was 0.13 ± 0.06 mm³ for the indirect group and 0.12 ± 0.08 mm³ for the direct group. The Mann–Whitney *U* test demonstrated no statistically significant difference in volumetric wear between the two groups (*U* = 45.00, *p* = 1.0).

## Discussion

This study was designed to compare the wear of opposing enamel in response to two widely used restorative materials for posterior overlays: milled resin-matrix ceramic and direct bulk-fill resin composite. A two-year evaluation period was chosen to allow adequate time for clinically meaningful wear changes to manifest, as shorter durations may not fully capture the long-term behavior of restorative materials in the oral environment. This timeframe also balances patient compliance with the need for longitudinal observation. These materials were selected due to their widespread clinical use and their differing compositions and mechanical properties, which were hypothesized to influence their interaction with opposing enamel.

Loss of tooth structure affects chewing ability, dental sensitivity, and appearance. It can also result in alterations in the occlusion. The need for teeth to last longer due to ageing populations has made the problem of tooth wear more pressing. The development of better restorative materials can be aided by an understanding of the causes behind tooth deterioration. As a result, a thorough evaluation of the tribological performance of restorative materials is crucial. Therefore, a thorough analysis of the elements influencing the wear mechanisms and the impact of various restorative materials on enamel wear is required [12].

Resin Matrix ceramics (RMCs) are a relatively new technology. The beneficial qualities of polymers and ceramics are combined in these materials. They have been referred to as “nano-ceramics” or “hybrid ceramics,” although these names are merely intended for commercial purposes and do not fully reflect the material’s physical and chemical makeup. RMCs have better flexural strength, machinability, and edge stability than ceramics because they contain both ceramic and polymer phases. They are also less brittle. High-temperature polymerized resin-based composites (RBCs) with dispersed ceramic fillers and high-temperature/high-pressure polymer-infiltrated ceramic network (PICN) are two further classifications for RMCs based on their industrial polymerization mechanism and microstructure. The strongly cross-linked polymeric matrix of CAD/CAM RBCs is reinforced by nano or nano-hybrid ceramic fillers, and the organic phase predominates [13].

Light-polymerized composite resins have advantages over dual-polymerized resin types of cement that include stain resistance, color stability, and mechanical wear resistance because of increased inorganic filler loading. The high inorganic filler content directly influences the viscosity of the composite resin, making it less fluid and leading to a thicker, undesirable, cementation line at the adhesive interface. As an alternative to reduced viscosity luting agents, the preheating of composite resins has been suggested. By increasing the temperature from 54 °C to 70 °C, the degree of conversion of the resin becomes similar to that of dual-polymerizing resin cement, the consistency of flow improves, and a thinner cementation line becomes possible. The preheated composite resin used in cementation resulted in the better seating of inlays, onlays, and overlays than the dual-polymerizing resin cement [14].

Intraoral 3D scanning is increasingly available in general dental practices, providing advantages for both patients and clinicians by enhancing efficiency, convenience, and ease of data storage and sharing. By superimposing scans of teeth or jaws, 3D data enables precise measurement of wear by quantifying differences between scans. This method allows for an objective assessment of tooth material loss in terms of height or volume. However, achieving highly accurate quantitative wear analysis often requires advanced techniques to ensure precise measurements [15]. To ensure the accuracy and reliability of alignment and deviation analysis, metrology-grade software was utilized in this study. Unlike standard dental CAD software, metrology-grade programs are specifically designed for high-precision measurement and analysis, offering advanced algorithms for surface registration and deviation quantification. These tools employ iterative best-fit methods, minimizing overlay errors and enhancing the robustness of superimposition. Additionally, metrology software provides superior noise filtering and compensation for potential inaccuracies inherent in intraoral scans, ensuring that deviations are accurately detected and quantified. By using such high-precision software, the study aimed to achieve consistent and reproducible results, reducing variability in wear assessment and enhancing the validity of patient monitoring over time. The volumetric measurement technique employed in this study aligns with the methodology outlined in previous research, where similar principles were applied to assess occlusal wear using superimposed 3D datasets [16].

According to the results obtained from the current study, no statistically significant difference between the wear amount of opposing enamel to BRILLIANT Crios and Filtek™ One Bulk Fill overlays measured by the intraoral scanner and Geomagic Control × 3D superimposition software. Therefore, the null hypothesis was confirmed.

Enamel wear was observed to increase as the hardness of composite resins increased. Additionally, composite resins became more resistant to wear relative to human enamel with greater hardness. This suggests that the degree of enamel wear is influenced by the filler particles’ characteristics specifically their size, shape, hardness, and concentration. As a result, a clear correlation exists between these filler properties and the overall hardness and wear resistance of the composites. Notably, it was also found that when a composite resin’s hardness exceeds 45 Vickers Hardness Number (VHN), its wear resistance surpasses that of enamel, potentially causing wear on the opposing enamel surface [17].

Based on previous studies [18, 19] Filtek™ One Bulk Fill demonstrated a hardness of approximately 70 VHN, while BRILLIANT Crios showed a slightly higher value of around 77 VHN. Given the close similarity in their hardness levels, there was no significant difference observed in the amount of wear they caused to the opposing enamel.

However, clinical data on the wear behavior of natural teeth remain limited, and the existing evidence shows significant variation. Reported wear rates for natural enamel in the few available studies range between approximately 10 and 40 μm per year. Similarly, only a small number of studies have assessed the in vivo wear of posterior composite crowns, with average wear rates reported around 40 μm per year [3]. These results are consistent with the findings observed in our study. This suggests that both direct and indirect composite materials are compatible with opposing enamel, as the wear they produce falls within the range of natural physiological enamel wear.

Resin composite indirect restorations, such as overlays and onlays, are a good substitute for a conservative and adhesive method. Resin composites are also appropriate as a minimally invasive treatment because they can be applied directly. These crucial features make them the perfect materials to treat patients with parafunctions and occlusal problems, as well as extended occlusal rehabilitations and entire mouth rehabilitations. Easy handling qualities and a reasonably modest cost are further benefits. Additionally, they enable simple intraoral changes for appropriate occlusion and efficient surface polishing based on real clinical needs, potentially cutting down on patient chair time [20].

This study utilized an intraoral scanner to quantitatively assess wear on the occlusal surfaces of restorations. However, the virtual models produced by the scanner are not entirely free from defects, and the inherent limitations of digitalization may affect the effectiveness of patient monitoring when comparing models. Additionally, the results are highly dependent on software parameters and the accuracy of reference area superimposition. To ensure the reliability of clinical trials and prevent misleading variations, it is crucial to capture both the initial and final models under consistent environmental conditions. Furthermore, saliva can influence outcomes and reduce the accuracy of intraoral scanning. To minimize this effect in the present study, the restored teeth were isolated using a lip retractor, dry pads, and curly saliva ejectors before scanning, following the manufacturer’s guidelines. The quantitative measurement method is not specific to the regions opposing the restorations exclusively thus the measurement is a combined effect of normal physiologic tooth wear and restoration mediated wear.

## Conclusions

Within the limitations of this two-year clinical evaluation, no statistically significant differences were observed between direct bulk-fill resin composites and milled resin matrix ceramic overlays in terms of their interaction with opposing enamel in posterior teeth.

## Data Availability

The data that support the findings of this study are available from the corresponding author on reasonable request.
